# Whole Body Low Dose Computed Tomography (WBLDCT) Can Be Comparable to Whole-Body Magnetic Resonance Imaging (WBMRI) in the Assessment of Multiple Myeloma

**DOI:** 10.3390/diagnostics11050857

**Published:** 2021-05-11

**Authors:** Davide Ippolito, Teresa Giandola, Cesare Maino, Davide Gandola, Maria Ragusi, Pietro Andrea Bonaffini, Sandro Sironi

**Affiliations:** 1 Department of Diagnostic Radiology, “San Gerardo” Hospital, via Pergolesi 33, 20900 Monza, MB, Italy; teresagiandola@hotmail.it (T.G.); mainocesare@gmail.com (C.M.); gandolad@gmail.com (D.G.); maria.ragusi@gmail.com (M.R.); 2 School of Medicine, University of Milano-Bicocca, via Cadore 48, 20900 Monza, MB, Italy; pa.bonaffini@gmail.com (P.A.B.); sandrosironi@libero.it (S.S.); 3 Department of Diagnostic Radiology, H Papa Giovanni XXIII, Piazza OMS 1, 24127 Bergamo, BG, Italy

**Keywords:** multiple myeloma, multidetector computed tomography, hematologic neoplasms, radiation dosage, osteolysis, magnetic resonance imaging, diffusion-weighted imaging

## Abstract

Aim of the study is to compare the agreement between whole-body low-dose computed tomography (WBLDCT) and magnetic resonance imaging (WBMRI) in the evaluation of bone marrow involvement in patients with multiple myeloma (MM). Patients with biopsy-proven MM, who underwent both WBLDCT and WBMRI were retrospectively enrolled. After identifying the presence of focal bone involvement (focal infiltration pattern), the whole skeleton was divided into five anatomic districts (skull, spine, sternum and ribs, pelvis, and limbs). Patients were grouped according to the number and location of the lytic lesions (<5, 5–20, and >20) and Durie and Salmon staging system. The agreement between CT and MRI regarding focal pattern, staging, lesion number, and distribution was assessed using the Cohen Kappa statistics. The majority of patients showed focal involvement. According to the distribution of the focal lesions and Durie Salmon staging, the agreement between CT and MRI was substantial or almost perfect (all κ > 0.60). The agreement increased proportionally with the number of lesions in the pelvis and spine (κ = 0.373 to κ = 0.564, and κ = 0.469–0.624), while for the skull the agreement proportionally decreased without reaching a statistically significant difference (*p* > 0.05). In conclusion, WBLDCT showed an almost perfect agreement in the evaluation of focal involvement, staging, lesion number, and distribution of bone involvement in comparison with WBMRI.

## 1. Introduction

Multiple myeloma (MM) is a monoclonal plasma cell proliferative disorder characterized by primary bone marrow infiltration and excessive production of abnormal monoclonal immunoglobulin [1].

Up to 90% of patients with MM develop bone lesions during illness course, underlining the importance of imaging examinations at the time of diagnosis and during follow-up, especially considering that number and size of focal bone lesions have been shown to predict outcome [2,3].

The diagnosis of MM mainly relies on the demonstration of bone marrow plasmacytosis and/or presence of monoclonal proteins (M-proteins) in the serum or urine, and/or detection of end-organ damage (CRAB—hypercalcemia, renal failure, anemia, and bone disease), especially lytic bone lesions, based on the International Myeloma Working Group (IMWG) diagnostic criteria published in 2014 [1,4,5,6]. Indeed, at imaging assessment, the evidence of at least two 5-mm or larger focal lesions detected at magnetic resonance imaging (MRI) is considered to be a myeloma biomarker, while at least one 5-mm or larger osteolytic lesion at positron emission tomography (PET), whole-body low dose computed tomography (WBLDCT), or whole-skeletal x-ray is considered to be a CRAB feature [7].

Cross-sectional imaging is preferred over conventional radiography (CR) for the evaluation of bone disease because of higher sensitivity [1,8,9,10,11,12,13,14] but the choice of imaging modality is made according to availability, cost, and institutional preference [15].

Whole-body MRI (WBMRI) is becoming increasingly relevant for the assessment of patients with MM, due to complete body coverage, excellent sensitivity for bone marrow involvement before/without bone destruction (i.e., in case of a diffuse pattern), and availability of advanced techniques such as diffusion-weighted imaging (DWI) and Dixon-based fat-fraction evaluation [16,17,18]. Moreover, MRI offers the additional benefits of assessing common MM complications, such as spinal canal and nerve root compression, and is the most accurate technique to differentiate benign and malignant vertebral compression fractures [19]. Hence, WBMRI is now recommended as first-line imaging modality for all patients with a suspected diagnosis of asymptomatic myeloma or solitary bone plasmacytoma and, in the United Kingdom, for all patients with a suspected new diagnosis of MM [20,21]. Nevertheless, many issues regarding availability, cost standardization, and radiologic expertise prevent WBMRI from becoming more widely accepted as the imaging modality of choice in MM patients’ management, including initial assessment.

On the other hand, there are several advantages in the use of WBLDCT as a first-line imaging modality in newly diagnosed patients with MM: wide availability, less expensive cost (compared to other cross-sectional imaging modalities), and quickness (acquisition time of about 30–40 s) [22]. Besides, WBLDCT has a sensitivity of 70% and a specificity of 90%, with MRI as the reference standard [23], and it offers the advantage of both whole-body coverage and low effective radiation dose delivered to the patient [10,11,12,24,25,26]. Moreover, according to a recent study, WBLDCT represents a useful imaging modality to evaluate not only focal lytic lesions but also diffuse bone involvement analyzing the peripheral medullary patterns of attenuation [27]. According to the European Myeloma Network (EMN) [28] and the European Society for Medical Oncology (ESMO) [29], WBLDCT is recommended as the initial reference standard procedure for the diagnosis of lytic bone disease in patients with MM.

On this basis, this study aims to compare the agreement between WBLDCT and WBMRI in the evaluation of bone marrow involvement in patients with MM in different stages of the disease.

## 2. Materials and Methods

The Local Ethics Committee’s review of the protocol deemed that formal approval was not required owing to the retrospective, observational, and anonymous nature of this study.

We retrospectively enrolled patients with biopsy-proven MM diagnosed between January 2008 and November 2019.

Inclusion criteria were: (1) age > 18 years, (2) diagnosis of MM according to the International Myeloma Working Group, (3) having undergone at least a WBMRI and a WBLDCT examination for staging purposes or during the follow-up of disease, and (4) a maximum 6 months interval between the two diagnostic exams.

**Figure 1:**
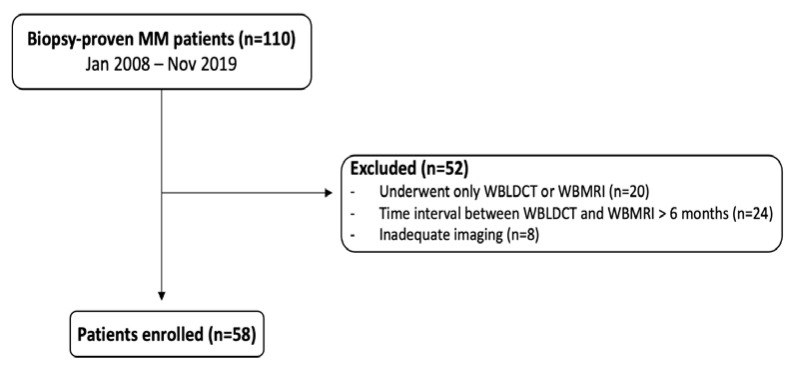
Flow chart of the study.

The exclusion criteria were: (1) technical inadequate examinations and (2) diffuse involvement of the disease. Flow-chart in Figure 1 summarizes the study design.

### 2.1. WBLDCT Protocol

Unenhanced WBLDCT studies were obtained using a 256-slice scanner (iCT, Philips) with the following protocol: tube voltage 120 kV, tube current-time product 40 mAs, collimation 128 × 0.65, pitch 1, gantry rotation time 270 ms, acquisition time 10–15′′. Patients were positioned supine and headfirst, with arms beside the body to allow evaluation of the upper limbs and slightly displaced in the anterior direction to avoid beam-hardening artifacts in the spine. The field of view (FOV) was adapted to body habitus, including a total body volume from the roof of the skull to the proximal tibial metaphysis. Images were acquired in inspiratory apnoea during the scanning through the thorax and the upper abdomen. Raw data were reconstructed with a slice thickness of 2 mm and an increment of 1 mm. In cases of a metal prosthesis, the metal artifacts reduction algorithm (O-MAR) was used. WBLDCT protocol is summarized in Table 1.

The dose report, expressed as CT dose index (CTDI) and dose–length product (DLP) were recorded from the examination summary reports produced by the CT scanner for each patient, while the effective dose (ED) was calculated using methods previously reported [14].

### 2.2. WBMRI Protocol

Whole body-MRI examinations were performed on a 1.5 T magnet (Ingenia, Philips, The Netherlands) with the following protocol: T1-weighted turbo spin-echo (TSE) and T2-weighted short Tau inversion recovery (STIR) sequence acquired on the coronal and sagittal planes from the skull vertex to the feet; diffusion-weighted imaging with background suppression (DWIBS) sequences acquired on an axial plane with 3 b-values (0, 500, and 1000).

All the study sequences were acquired during free breathing, with a slice thickness of a 4-mm and 1-mm gap. The total acquisition time was about 45–55 min, depending on the patient’s height. At the end of the study, every imaged district was merged using software integrated with the scanner, generating coronal whole-body T1, T2 STIR, spinal sagittal T1, T2 STIR, and DWIBS reconstructions.

The patient was positioned supine headfirst, using two-phased body-array coils, inline, for the examination of the thorax, abdomen, pelvic region and upper and lower limbs, and one head-and-neck coil for the head and neck regions. All WBMRI studies were performed in the stepping-table movement technique. In the case of a metal prosthesis, the metal artifacts reduction sequences (MARS), both T1- and T2-weighted, were used. WBMRI protocol is summarized in Table 2.

### 2.3. Image Analysis

All WBLDCT and WBMRI images were evaluated, to identify signs of bone involvement, by a single radiologist with 15 years of experience in MM CT and MRI imaging.

Images were evaluated in terms of infiltration pattern (focal, diffuse, and combined) in the comparison between WBLDCT and WBMRI, only focal involvement was considered on both techniques, due to the lower sensitivity of WBLDCT, when compared to WBMRI, in the evaluation of diffuse bone marrow infiltration, making it difficult to differentiate myeloma-related osteopenia from osteoporosis [7]. Moreover, up to date, among all known MM infiltration patterns, the focal involvement is the only one reported to affect the disease stage according to international guidelines [1].

On WBLDCT, the diagnosis of osteolytic bone lesions was performed analyzing both axial and multiplanar reformatted (MPR) images: the typical, punched-out lytic lesions with a diameter of 5 mm or more were recorded as focal myeloma involvement, according to the recent guidelines [6]. The assessment of spinal involvement was performed using MPR images, especially on the sagittal plane.

On WBMRI, the diagnosis of bone marrow involvement was performed using mainly T1-weighted TSE and fat-suppressed sequences (STIR). Pathological replacement of bone marrow leads to a typical modification of the signal intensity with a focal decrease in the signal intensity on T1-weighted TSE corresponding to a focal increase in the signal intensity on T2-weighted STIR images. The DWIBS sequences were used to confirm the presence of focal bone involvement recorded considering that pathologic bone marrow usually exhibits restricted diffusion, with a higher signal on high b-value compared to the very low signal of normal bone marrow.

Moreover, to better evaluate the diagnostic efficacy of both imaging techniques, according to different anatomic regions, the whole skeleton was divided into five anatomic districts: (1) skull, (2) spine, (3) sternum and ribs, (4) pelvis, and (5) upper and lower limbs.

All the typical focal bone lesions were recorded based on their location and for each anatomic district the number of the lytic lesions was recorded according to three subgroups: (1) less than 5, (2) between 5 and 20, and (3) more than 20 lesions.

Furthermore, patients were evaluated according to the Durie and Salmon PLUS staging system (24) as follows: Stage IA if present limited disease at imaging or single plasmacytoma, Stage IB if present less than 5 focal lesions, Stage II if present between 5–20 focal lesions, and Stage III if present more than 20 focal lesions.

### 2.4. Statistical Analysis

Categorical data were expressed in numbers and percentages, while continuous ones in mean and standard deviation (SD) or median and interquartile range (IQR), as appropriate. The agreement between CT and MRI was assessed using the Cohen Kappa coefficient (0.00–0.20 indicates slight agreement; 0.21–0.40, fair agreement; 0.41–0.60, moderate agreement; 0.61–0.80, substantial agreement; and 0.81–1.00, almost perfect agreement). Bias-corrected 95% CI was computed using bootstrap with 1000 iterations. A *p*-value < 0.05 was considered significant. The analysis was performed using SPSS software (v 26.0, SPSS Inc., Chicago, IL, USA).

## 3. Results

### 3.1. Patients Population

By applying the aforementioned inclusion and exclusion criteria, the final cohort consisted of 58 patients, as summarized in the flow chart in Figure 1, the majority female (M/F = 28/30), with a mean age of 69 (±9) years.

### 3.2. CT Findings

According to the infiltration pattern, 35/58 (60%) patients showed a focal involvement. Overall, the most common involved anatomic district was spine (*n* = 29, 50%), followed by pelvis (*n* = 20, 34.5%), and skull (*n* = 18, 31%). The majority of patients showed less than 5 lesions (*n* = 48, 83%), while 39 (67%) and 4 (7%) from 5 to 20 and more than 20 lesions, respectively, as reported in Table 3. A total of 23 patients (39.7%) were judged as negative. According to the Durie and Salmon staging system, the majority of patients were classified in stage IB and II, 29 (50%) and 11 (19%), respectively, as reported in Table 4.

The mean DLP value was 464.6 mGy*cm (±121), CTDI 2.7 mGy (±0.4), and ED 3.9 mSv (±0.9).

### 3.3. MRI Findings

**Figure 2:**
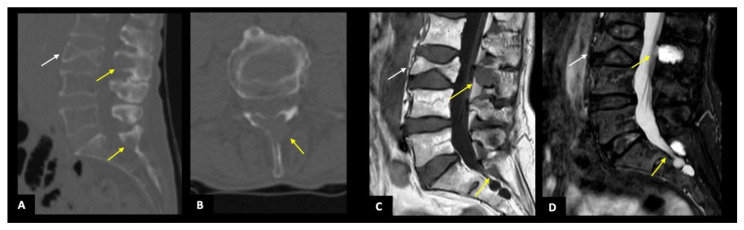
A 67-year-old man with a focal infiltration pattern (**A**) MPR image in a sagittal view showing some typical lytic lesions localized in the spinous processes of L3 and S1 (yellow arrows) and a vertebral fracture of L3 (white arrow). The sagittal plane allows a proper assessment of the regular alignment of posterior vertebral bodies. (**B**) CT axial section of L3 showing a lytic lesion with focal cortical interruption of the spinous process. The same bone lesions in the spinous processes of L3 and S1 are visible in images (**C**) as areas of hypointensity in the T1-weighted sequence of the spine (yellow arrows) and (**D**) as areas of hyperintensity in STIR-weighted sequence of the spine (yellow arrows). The vertebral collapse of L3 has confirmed both in (**C**,**D**) images (white arrow) but better dated as non-recent (no evidence of edema on STIR sequence).

According to the infiltration pattern, 36/58 (62.1%) showed a focal involvement; among them, 23 (39.7%) presented a focal pattern while 13 (22.4%) a combined one (focal involvement associated with a diffuse bone marrow infiltration). Overall, the most common involved anatomic district was the spine (*n* = 31, 53%) (Figure 2), followed by pelvis (*n* = 17, 29%), and limbs, both upper and lower (*n* = 15, 26%). The majority of patients showed less than 5 lesions (*n* = 54, 93%), while 26 (45%) and 2 (3%) from 5 to 20 and more than 20 lesions, respectively, as reported in Table 3. A total of 11 patients (19%) were judged as negative. According to the Durie and Salmon staging system, the majority of patients were classified in stage IB and II, 30 (52%), and 12 (21%), respectively, as reported in Table 4.

### 3.4. Imaging Agreement

According to distribution, the agreement between CT and MRI was substantial for focal involvement pattern (κ = 0.875, *p* < 0.0001), while CT was judges more frequently as negative in comparison with MRI (*n* = 23 and *n* = 11, κ = 0.459 and *p* < 0.0001), as reported in Table 4.

According to the Durie and Salmon staging system, CT and MRI showed an almost perfect agreement in subgroups IA and III (κ = 0.716 and κ = 0.815, *p* < 0.0001, respectively), while a significant fair agreement was found in subgroups IB and II (0.41< κ < 0.60).

**Figure 3:**
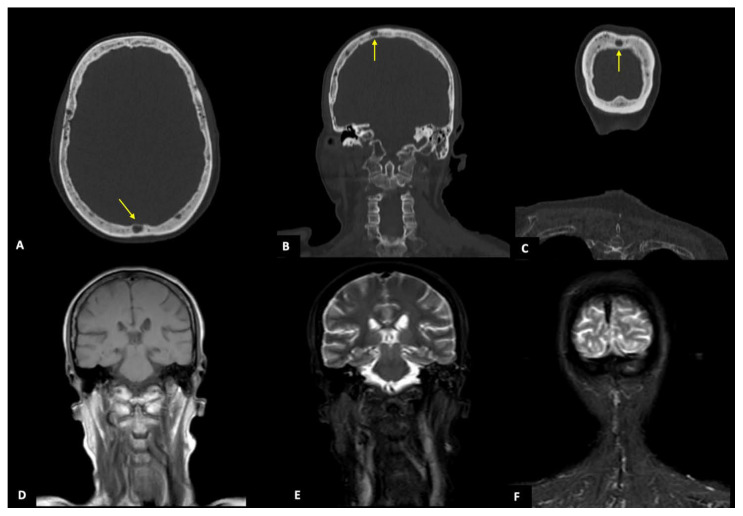
CT and MRI images from a 64-year-old woman with a focal infiltration pattern. (**A**–**C**): WBLDCT axial section and MPR images in coronal view showing some focal lytic lesions of the skull of whom the biggest (15 mm) is highlighted by the yellow arrow. The bone lytic lesions are not recognizable at all in image (**D**), a coronal T1-weighted image of the skull nor in images (**E**,**F**), and coronal STIR-weighted images of the skull.

**Figure 4:**
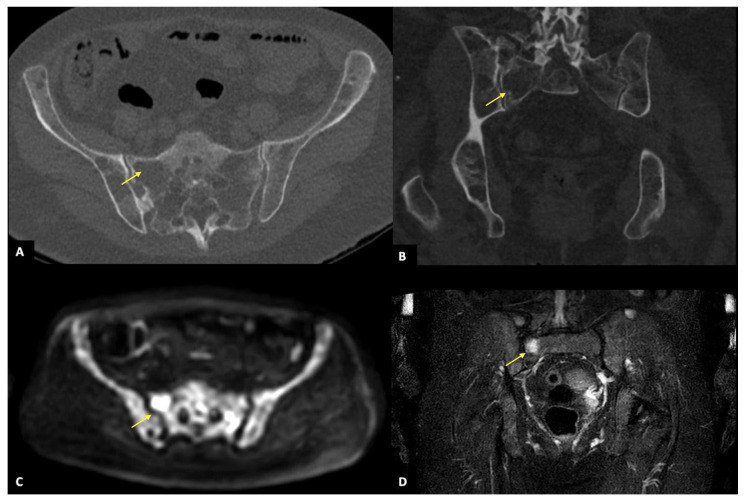
A 76-year-old man with a focal infiltration pattern showing numerous bone lesions in the pelvis, of whom the biggest one localized in the right side of the sacrum (yellow arrow) and recognizable in both imaging techniques. (**A**,**B**): WBLDCT axial view and MPR image in the coronal plane showing the bone lesion in the right side of the sacrum (yellow arrow) as a lytic lesion that partially erodes the cortical bone. The same lesion is visible in image (**C**) as a focal area of diffusion restriction (yellow arrow) in axial DWIBS sequence with 800 b-value and in image (**D**) as a focal area of hyperintensity (yellow arrow) in the coronal STIR-weighted sequence. On DWIBS there is also evidence of diffusely increased signal intensity in the background, as in cases of the combined pattern.

**Figure 5:**
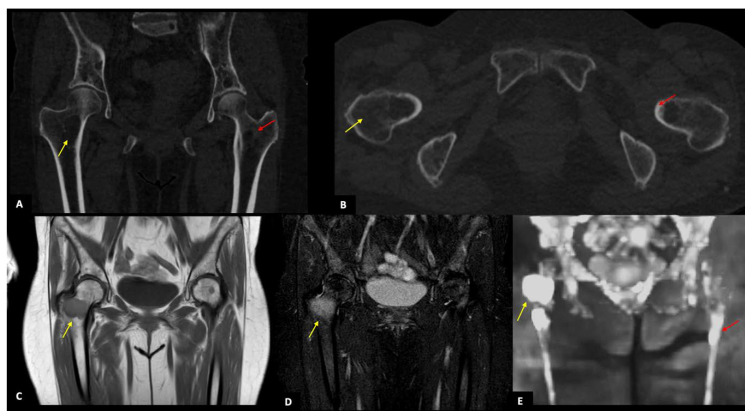
A 68-year-old woman with a focal infiltration pattern. (**A**,**B**): WBLDCT MPR image in coronal and axial planes showing some focal lytic lesions of which the two biggest are localized in the right neck of femur (yellow arrow, 44 mm) and the left neck of femur (red arrow, 23 mm), respectively. (**C**,**D**) Coronal T1 and STIR-weighted sequences showing the same lytic lesions of the right neck of femur (yellow arrow) as areas of hypointensity in (**C**) and hyperintensity in (**D**) The reconstructed 3D DWIBS sequence (**E**) shows the bone lesions described before, in the neck of femur bilaterally, as areas of hyperintensity (yellow and red arrows).

According to anatomic districts, the overall agreement between the two imaging techniques was almost perfect for upper and lower limbs (κ = 0.820), and substantial regarding sternum, ribs, and spine (κ = 0.644 and κ = 0.586, respectively), while a fair and slight agreement was found for pelvis and skull (κ = 0.486 and κ = 0.283) (Figure 3 and Figure 4). By subgrouping patients according to lesion number (<5, 5–20, and >20), we confirmed that the agreement remained significantly almost perfect regarding upper and lower limbs (Figure 5), sternum, and ribs (all κ > 0.6). Interestingly, regarding pelvis and spine, the agreement increased proportionally with the number of lesions (κ = 0.373–0.564, and κ = 0.469–0.624, if <5 and between 5 and 20 lesions, respectively, all *p* < 0.05) (Figure 2 and Figure 4), while for the skull the agreement proportionally decreased without reaching a statistically significant difference. All agreement values are reported in Table 5.

## 4. Discussion

In our series, WBLDCT and WBMRI imaging were used to analyze 58 MM patients with focal bone marrow involvement. In both techniques, the majority of patients showed skeletal involvement by myelomatous lytic lesions with a focal infiltration pattern, in line with the previous paper [30].

In 81% of cases, the focal infiltration pattern of disease was concordant in both WBMRI and WBLDCT achieving an almost perfect agreement between the two techniques.

The agreement between WBLDCT and WBMRI was also evaluated according to the Durie and Salmon PLUS staging system. In 76% of cases, the diagnostic techniques were concordant regarding the stage of the disease reaching a statistically significant agreement. This was particularly true for patients with stage IA, who had no bone lesions, and for those with stage III, who had more than 20 bone lytic lesions.

These results, therefore, highlighted that WBMRI and WBLDCT are both reproducible and reliable imaging techniques not only in the analysis of bone involvement but also in the evaluation of the prognosis of patients with multiple myeloma according to their staging.

When analyzing the anatomic distribution of myelomatous lesions within the focal and combined pattern of disease, our results showed that, in both techniques, the bone lytic lesions were predominantly distributed in the spine and the pelvis, in line with previous papers [31,32,33].

Furthermore, WBMRI and WBLDCT showed a statistically significant concordance in the detection of the bone lesions according to the number and anatomic distribution, in particular in the sternum, ribs, and upper and lower limbs.

Although the strong agreement in the detection rate of MRI and CT, considering the skull, the concordance between the two techniques decreased proportionally with the number of lesions. This result, therefore, underlined that WBMRI was significantly less sensitive than WBLDCT in the detection of bone lesions of the skull, probably because of the small size of lesions and the poor signal intensity of the skull itself, due to its intrinsic thickness and presence of bone–air interface. Quite the opposite, WBLDCT is particularly sensitive to recognize the classic “oil droplet” appearance of the skull, characterized by innumerable small lytic lesions with uniform size.

MRI remains the most sensitive and specific imaging method for the detection of bone marrow infiltration before the mineralized bone has been destroyed [34] and it is recommended by the British Society of Haematology for monitoring the response of non-secretory or oligosecretory myeloma and for those patients with the extramedullary disease [35]. According to the results of several studies [36,37,38,39], WBMRI is even more sensitive than PET/CT for the detection of focal or diffuse bone involvement and a large number of focal lesions. Therefore, MRI is considered the reference standard method for bone marrow assessment.

The IMWG recommended WBLDCT as the first-line imaging modality in suspects of MGUS, smoldering MM, and multiple myeloma, and cases of suspected relapse of disease [39]. Moreover, the ESMO and EMN guidelines advocate the use of WBLDCT as a new standard for detecting relevant osteolytic lesions [28,29]. WBLDCT is indeed fast, less cumbersome for patients, relatively sensitive and cost-effective, with an excellent interobserver correlation and a low radiation dose delivered to patients, as previously reported [15,40].

Our results highlighted an excellent concordance between both techniques, suggesting their potential interchangeability in the evaluation of focal bone marrow involvement in patients with MM, in particular considering the short time interval between WBLDCT and WBMRI considered in our analysis.

However, some limitations should be noted, first of all, the retrospective design of the study and secondly, the small size of the cohort studied, due to the strict inclusion criterion regarding time interval between CT and MRI. Finally, only one radiologist assessed the WBLDCT and WBMRI images, and, consequently, the interobserver agreement was not computed.

## 5. Conclusions

In conclusion, WBLDCT showed an almost perfect agreement in the evaluation of focal bone involvement, staging, lesion number, and distribution of bone involvement in comparison with WBMRI.

## Figures and Tables

**Table 1:** WBLDCT protocol.

WBLDCT Parameter	Measurement
Scanner	256-slice scanner
Scan coverage	Cranial vault to the distal tibial metaphysis
Tube voltage (kV)	120
Tube current-time product (mAs)	40
Collimation (mm)	128 × 0.65
Pitch	1
Thickness/Increment of axial slices (mm)	2/1
Gantry rotation time (ms)	270
Acquisition time (s)	10–15

**Table 2:** WBMRI protocol. (TSE: turbo spin echo; STIR: short tau inversion recovery; DWIBS: diffusion weighted imaging with background suppression; TE: echo time; TR: repetition time; DFOV: display fold of view; SNR: signal-to-noise ratio. * b-values: 0–500–1000).

Sequence	Plane	Coverage	TE (ms)	TR (ms)	DFOV (mm)	Voxel Size (mm)	Section Thickness (mm)	SNR
T1-TSE	Coronal	Vertex to feet	15	922	365	1.16 × 1.46	6	1.00
T2-STIR-TSE	Coronal	Vertex to feet	60	8704	365	1.25 × 1.82	6	1.00
T1-TSE	Sagittal	Whole spine	7.4	408	270	0.90 × 1.15	3.5	1.00
T2-STIR-TSE	Sagittal	Whole spine	60	2533	270	0.90 × 1.25	3.5	1.00
DWIBS *	Axial	Vertex to feet	66	6421	520	5.00 × 4.98	6	1.00

**Table 3:** Lesion distribution in different anatomic districts on CT and MRI, according to number (categories <5, 5–20, and >20).

District (*n*, %)	Number of Detectable Lesions					
	CT			MRI		
	<5	5–20	>20	<5	5–20	>20
Skull	13 (22.4)	4 (6.9)	1 (1.7)	4 (6.9)	1 (1.7)	0 (0)
Sternum and ribs	5 (8.6)	6 (10.3)	0 (0)	11 (19)	3 (5.2)	0 (0)
Pelvis	14 (24.1)	6 (10.3)	0 (0)	13 (22.4)	4 (6.9)	0 (0)
Spine	13 (22.4)	13 (22.4)	3 (5.2)	14 (24.1)	15 (25.9)	2 (3.4)
U/L limbs	3 (5.2)	10 (17.2)	0 (0)	12 (20.7)	3 (5.2)	0 (0)

**Table 4:** Distribution pattern and Durie and Salmon stage according to CT and MRI findings. The agreement was reported as the κ value and relative 95% CIs.

Pattern (*n*, %)	CT	MRI	Agreement (κ; 95% CI)	*p*-Value
No detectable lesions	23 (39.7)	11 (19.0)	0.459 (0.351–0.699)	<0.0001
Focal involvement	35 (60.3)	36 (62.0)	0.875 (0.783–0.951)	<0.0001
Diffuse	-	11 (19.0)		
Combined	-	13 (22.4)		
Durie Salmon Stage (*n*, %)				
IA	9 (15.5)	11 (19)	0.759 (0.473.0.949)	<0.0001
IB	29 (50)	30 (51.7)	0.552 (0.345–0.724)	<0.0001
II	11 (19)	12 (20.7)	0.512 (0.186–0.776)	0.001
III	9 (15.5)	5 (8.6)	0.772 (0.473–1.000)	<0.0001

**Table 5:** Agreement between CT and MRI findings according to anatomic districts and number of lesions. The agreement was reported as the κ value and relative 95%CI. * *p*-value < 0.0001, ^ *p*-value < 0.05, ° *p*-value > 0.05, § not computed.

District	Agreement (κ; 95%CI)			
	Overall	<5 Lesions	5–20 Lesions	>20 Lesions
Skull	0.283 (0.056–0.510) *	0.145 (0.010–0.415) °	0.028 (−0.069–0.100) °	§
Sternum and ribs	0.644 (0.405–0.883) *	0.433 (0.103–0.789) *	0.642 (0.180–0.990) *	§
Pelvis	0.486 (0.247–0.725) ^	0.373 (0.092–0.665) ^	0.564 (0.024–0.844) *	§
Spine	0.586 (0.379–0.793) ^	0.469 (0.196–0.707) *	0.624 (0.372–0.834) *	0.791 (0.122–0.993) *
U/L limbs	0.820 (0.619–0.961) *	0.776 (0.518–0.949) *	0.733 (0.710–0.783) *	§
